# The activation of Proteinase-Activated Receptor-1 (PAR1) mediates gastric cancer cell proliferation and invasion

**DOI:** 10.1186/1471-2407-10-443

**Published:** 2010-08-19

**Authors:** Daisuke Fujimoto, Yasuo Hirono, Takanori Goi, Kanji Katayama, Shigeru Matsukawa, Akio Yamaguchi

**Affiliations:** 1 First Department of Surgery, Faculty of Medicine and Division of Bioresearch Laboratories, University of Fukui, Fukui, 910-1193, Japan; 2 Centers for Advanced Research Support, University of Fukui, Fukui, 910-1193, Japan

## Abstract

**Background:**

In addition to regulating platelet function, the G protein-coupled sub-family member Proteinase-activated receptor-1 (PAR1) has a proposed role in the development of various cancers, but its exact role and mechanism of action in the invasion, metastasis, and proliferation process in gastric cancer have yet to be completely elucidated. Here, we analyzed the relationship between PAR1 activation, proliferation, invasion, and the signaling pathways downstream of PAR1 activation in gastric cancer.

**Methods:**

We established a PAR1 stably transfected MKN45 human gastric cancer cell line (MKN45/PAR1) and performed cell proliferation and invasion assays employing this cell line and MKN28 cell line exposed to PAR1 agonists (α-thrombin and TFLLR-NH_2_). We also quantified NF-κB activation by electrophoretic mobility shift assay (EMSA) and the level of Tenascin-C (TN-C) expression in conditioned medium by ELISA of MKN45/PAR1 following administration of α-thrombin. A high molecular weight concentrate was derived from the resultant conditioned medium and subsequent cultures of MKN45/PAR1 and MKN28 were exposed to the resultant concentrate either in the presence or absence of TN-C-neutralizing antibody. Lysates of these subsequent cells were probed to quantify levels of phospholyrated Epidermal Growth Factor Receptor (EGFR).

**Result:**

PAR1 in both PAR1/MKN45 and MKN28 was activated by PAR1 agonists, resulting in cell proliferation and matrigel invasion. We have shown that activation of NF-κB and EGFR phosphorylation initially were triggered by the activation of PAR1 with α-thrombin. Quantitative PCR and Western blot assay revealed up-regulation of mRNA and protein expression of NF-κB target genes, especially TN-C, a potential EGFR activator. The suppressed level of phosphorylated EGFR, observed in cells exposed to concentrate of conditioned medium in the presence of TN-C-neutralizing antibody, identifies TN-C as a putative autocrine stimulatory factor of EGFR possibly involved in the sustained PAR1 activation responses observed.

**Conclusion:**

Our data indicate that in gastric carcinoma cells, PAR1 activation can trigger an array of responses that would promote tumor cell growth and invasion. Over expression of NF-κB, EGFR, and TN-C, are among the effects of PAR1 activation and TN-C induces EGFR activation in an autocrine manner. Thus, PAR1 is a potentially important therapeutic target for the treatment of gastric cancer.

## Background

A dysregulation of the coagulation cascade in the setting of human tumors has been recognized for over a century [1]. In particular, active thrombin has been found to play an important role in terms of tumor behavior, affecting a variety of cancer-related processes including invasion, metastasis and tumor cell growth [2,3]. In large part, thrombin initiates cellular effects by cleaving and thus activating a novel set of Proteinase-activated receptors (PARs 1 and 4; but not PAR2), that are members of the G-protein-coupled receptor (GPCR) superfamily [4-8]. Although able to activate PARs 1 and 4, thrombin is not able to activate PAR2, which is a target for trypsin [9]. PAR1 has been found to be instrumental in cell growth and invasion of tumor-derived cells [10,11]. In addition to regulating cell function by the PARs, thrombin may also affect cell function via the activation of metalloproteinase-2 (MMP2) [12]. Apart from serine proteinases that can activate PARs to affect cancer cell behavior, MMPs have for some time been known to be involved in cancer metastasis and invasion [13-17]. Surprisingly, MMP1 has been observed, like thrombin, to regulate invasion and tumorigenesis of breast cancer-derived cells by a process involving PAR1 [18], providing an important link between tumor generated metalloproteinases and PAR signaling. Additionally the existence of cross-talk between GPCR and EGFR signaling systems has been established in various cancer cells and has been found to promote cancer cell proliferation and migration through EGFR transactivation in colon cancer and renal cell carcinoma. MMPs are required by some GPCRs which suggest a possible role for MMPs in the PAR1 activation system as PAR1 is a subfamily of GPCR [19,20]. In prostate cancer-derived cells, PAR1 over-expression has also been documented and has been linked to PAR1-stimulated activation of NF-κB, with an increase in NF-κB-regulated inflammatory cytokines like IL-6 and IL-8 [21]. The exact role and mechanism of action of PAR1 in this process remains unclear.

In our previous work, using an immunohistochemical approach with gastric carcinoma tissue, we found that the expression of PAR1, along with a metalloproteinase known to activate PAR1 (MMP1) was associated with poorer prognosis, compared with expression-negative tumors [22]. In this study, we hypothesized that PAR1 would play an important role in gastric carcinoma cells. To test this hypothesis, we evaluated the impact of PAR1 activation in gastric cancer-derived cells. Our data show that the signaling pathways stimulated by PAR1 in the gastric cancer-derived cells mediate proliferation and invasion, and Tenascin-C (TN-C) might play an important role in this signaling pathways stimulated by PAR1.

## Methods

### Reagents

An antibody against PAR1 (clone WEDE15) was purchased from BECKMAN COULTER (Fullerton, CA, USA). Anti-TN-C was purchased from IBL (Gunma, Japan) and TN-C-neutralizing antibody (Clone BC24) [23] was from Sigma-Aldrich (St. Lois, MO, USA). Anti-Bcl-2, phospho-specific antibodies against EGFR (clone 20G3) and phosphotyrosyl-1173 EGFR (clone 9H2) were purchased from Upstate Biotech (Temecula, CA, USA). Anti-NF-κB-p50 and -p52 were from Santa-Cruz Biotechnology (Santa-Cruz, CA, USA). Anti-cIAP1 was from R&D systems (Minneapolis, MN, USA). Anti-GAPDH was from IMGENEX (San Diego, CA, USA). Human α-thrombin was purchased from Sigma-Aldrich (catalog #T1063). The selective PAR1 antagonist SCH79797 (catalog #1592) (IC50 = 70 nM) and PAR1 agonist TFLLR-NH_2 _(catalog #1464) were purchased from Tocris Bioscience (Anonmouth, UK) [24]. The NF-κB inhibitor Caffeic acid phenethyl ester (CAPE) (IC50 = 25 μg) was purchased from Biomol (Plymouth Meeting, PA, USA) [25].

### Cell Culture

The human gastric cancer cell lines, MKN28, MKN45 MKN74, NUGC2, NUGC3, and KATOIII cells were obtained from the Riken Cell Bank (Tsukuba, Japan). TMK-1 was a gift from Dr. S Fushida (Kanazawa University, Japan). Cells were cultured at 37°C in 5% CO_2 _in RPMI-1640 medium containing 10% fetal bovine serum (FBS). Cells were propagated by mechanical re-suspension using a scraper, without the use of trypsin.

### Reverse transcription-PCR and quantitative RT-PCR analysis

Total RNA was extracted from gastric cancer cells with ISOGEN reagent (NipponGene, Tokyo, Japan). Single-strand cDNA prepared from 3 μg total RNA using MMLV reverse transcriptase (GIBCO, Calabasas, CA, USA) with an oligo (dT)_14 _primer that was used as a template for reverse transcription-PCR (RT-PCR) or quantitative-PCR (qPCR). The following primer pairs were used: GAPDH/5'-GGGAGCCAAAAGGGTCATCATCT-3' and 5'-GACGCCTGCTTCACCACCTTCTTG-3'; and PAR1/5'-TGTGAACTGATCATGTTTATG -3' and 5'-TTCGTAAGATAAGAGATATGT -3'.

qPCR analysis was also done with a PCR mixture containing each primer and SYBR Green master mix (Qiagen, Hilden, Germany). The PCR primer pairs for the NF-κB target genes were custom made (Hokkaido System Science, Hokkaido, Japan). Each sample was examined in triplicate and the amounts of cDNAs were normalized with respect to those of a GAPDH internal control.

### Construction of PAR1 expression plasmid

A human PAR1 cDNA sequence was isolated by PCR from a NUGC3. We amplified the PAR1 cDNA using a primer set as follows: PAR1-CX for the 5' primer, GGGGATCCCGGCAGAGCCCGGGACAATG; and PAR1-DX for the 3' primer, GGGAATTCTCCCAGCAGTCCCTTTTCC. Both primers incorporated 5'-BamH1 and 3'-EcoR1 sites, respectively. We amplified the BamH1 and EcoR1 site-tagged full-length PAR1 fragments, and cloned them into a pcDNA3.1 (Invitrogen, Carlsbad, CA, USA). Positive clones (pcDNA3.1-PAR1) were isolated and validated by DNA sequencing. The sequence agreed with the Genebank record: NM001992.

### Established PAR1-expressing MKN45 stable cell line

MKN45 cells were transfected using LipofectAMINE2000 (Invitrogen) and pcDNA3.1-PAR1 (MKN45/PAR1) or pcDNA3.1-empty-vector alone (for MKN45/mock as the control). Individual G418 resistant (0.75 mg/ml) clones were picked and analyzed for PAR1 expression by RT-PCR and immunoblotting of total cell extract.

### Western blot

Total cell protein was extracted using RIPA buffer. Proteins in the lysate were resolved by SDS-PAGE using a 5-20% SuperSep gel (Wako, Osaka, Japan). The resolved proteins were transferred to nitrocellulose membrane. Protein bands were incubated with primary antibody overnight at 4°C. Signals were visualized by enhanced chemiluminescence according to the manufacturer's instructions (GE Healthcare, Buckinghamshire, UK).

### Cell growth analysis

To examine the in vitro cell growth rate, MKN45/mock, MKN45/PAR1 and MKN28 cells were seeded into 24-well plates at 1.0×10^4^cells/well. Various cultures were incubated for different periods of time while being exposed to one or more of the following: PAR1 agonists, α-thrombin and TFLLR-NH_2_, and PAR1 antagonist, SCH79797 and resultant growth rates were quantified. The level of pro-thrombin was 1-2 μM, and a concentration of active thrombin in the 10 nM range was almost certainly physiologically relevant [26]. The TRAP analogue, TFLLR-NH_2_, can selectively activate PAR1 at concentrations lower than 50 μM [27]. Thus, we selected an α-thrombin concentration of 15 nM and a TFLLR-NH_2 _concentration of 30 μM to determine if these enzymes would stimulate proliferation of MKN45/PAR1 and MKN28 cells. Cell numbers were counted with a hemocytometer at 24, 48, 72 and 96 hrs after seeding of cells.

### Cell invasion assay

In addition to establishing that the activation of PAR1 in a gastric carcinoma cell background can stimulate cell replication, we wished to evaluate the ability of PAR1 to stimulate cell invasion. Invasion of cells through matrigel was determined using a Transwell system (CHEMICON) as described previously [28]. α-thrombin was added at 15 nM, TFLLR-NH_2 _was added at 30 μM and SCH79797 was added at 35, 70, or 150 nM to the cells (0.5×10^6^cells/well) in the upper well containing serum-free medium. After the addition of fresh medium containing 10% FBS to the lower chamber, incubation was continued for 24 hr at 37°C. The cells on the underside of the membrane were stained and dissolved in 10% acetic acid for measurement of A_560 nm_. The A_560 nm _of the MKN45/mock, MKN28 and MKN45/PAR1 cells cultured under noted conditions were determined and compared using the A_560 nm _of MKN45/mock and MKN28 cultured under a PAR1 agonist-free condition as a baseline.

### Measurement of NF-κB Activation by Electrophoretic Mobility Shift Assay

MKN45/mock and MKN45/PAR1 were treated for 0.5, 1, 2, 6, 12, and 24 hr with 15 nM α-thrombin. Nuclear fractions were extracted from the cultured cells using NE-PER (PIERCE, Rockford, IL, USA). Assays were performed using an oligonucleotide with the NF-κB motif, 5'-AGTTGAGGGGACTTTCCCAGGC-3', which was labeled with biotin for chemiluminescence detection. Nuclear extracts of MKN45/PAR1 and MKN45/mock were isolated and a Gel mobility shift assay was performed by incubating each of the nuclear extracts with the labeled probe and competing oligonucleotides in binding buffer. The complex was resolved by electrophoresis on a 5-20% SuperSep gel (Wako) in 0.5× TBE buffer at 4°C, transferred to N^+^nylon membrane, and detected by streptavidin-HRP using a Lightshift chemiluminescence electrophoretic mobility shift assay (EMSA) kit (PIERCE). Super-shift reactions were run as described above with the exception that 2 μg of polyclonal anti-NF-κB-p50 and -p52 antibodies were used.

### Assays of Tenascin-C levels in conditioned medium by means of ELISA

The levels of high molecular-weight TN-C protein were determined using an ELISA kit (IBL) for the conditioned medium of MKN45/mock and MKN45/PAR1 at 3, 6, and 12 hr after the addition of 15 nM α-thrombin. The collected samples were concentrated by using VIVAspin (Vivascience, Stonehouse, UK) ultra-filtration units and incubated in 96-well ELISA plates for 1 hr at 37°C. After washing out unreacted antibody, HRP-conjugated anti-TN-C was added, followed by incubation for 30 min at 4°C, and the color intensity was determined at 450 nm. Results were calculated from the mean absorbance of duplicate wells.

### Assays of TN-C initiated phosphorylation of EGFR

MKN45/PAR1 was separately exposed to 15 nM α-thrombin for either 3 hr or 12 hr period. We then collected each separate conditioned medium and filtered it removing proteins with molecular weights lower than 200 kDa, including α-thrombin, and retained the high molecular weight protein concentrate. The concentrate then underwent two rounds consisting of a ten-fold dilution with PBS followed by filtration to isolate the same high molecular weights proteins. The level of α-thrombin in the resultant concentrates was estimated by means of SDS-PAGE and Western blot and was found to be about 90% less than the α-thrombin level of the initial cell cultures from which the concentrates were derived. Subsequent separate cultures of MKN45/PAR1 cells underwent a 6 hr exposure to one of either of the high molecular weight concentrates. The cultures exposed to the concentrates derived from initial cultures exposed to 15 nM α-thrombin for 12 hr were incubated for 6 hr either in the presence or absence of TN-C-neutralizing antibody (Clone BC24, 25 μg/ml). After incubation each culture exposed to concentrates was lysed, and the lysate subjected to SDS-PAGE, Western blotting and probing to quantify levels of phosphorylated EGFR.

## Results

### PAR1 mRNA is expressed in Gastric Cancer Cell Lines

**Figure 1:**
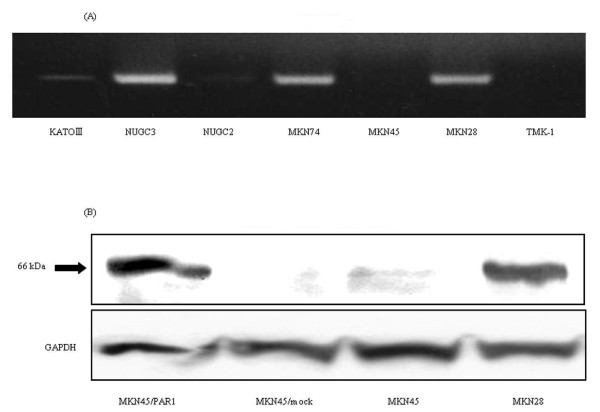
**The expression of PAR1 mRNA and protein in gastric cancer cell lines**. (A) The expression of PAR1 mRNA in 7 gastric cancer cell lines by RT-PCR. PAR1 mRNA was confirmed in MKN28, MKN74 and NUGC3. **(B) **Parent MKN45 cells and MKN45 cells transfected with pcDNA3.1-empty-vector alone (designated as MKN45/mock) have no detectable expression of PAR1 protein, and MKN28 and MKN45 cells transfected with PAR1 cDNA bearing pCDNA3.1 vector, which was treated 750 μg/ml G418 to select permanent PAR1 expressing clone, (designated as MKN45/PAR1) was confirmed to continuously express high level PAR1 protein by immunoblotting (upper panel). The bottom panel showed a protein band of a house keeping gene, GAPDH as a control. (The final dilution of PAR1 antibody was 1:500, GAPDH was 1:2000.)

The expression of PAR1 mRNA in seven gastric cancer cell lines was evaluated by RT-PCR and was found to be present in MKN28, MKN74, and NUGC3 cell lines (Figure 1A). Although, although a faint band was detected for KATOIII cells, no RT-PCR signal for PAR1 was detected for the NUGC2, MKN45, and TMK-1 cell lines (Figure 1A).

### Expression of PAR1 in MKN45 cells

Since MKN45 cells did not express PAR1, we selected this gastric carcinoma-derived cell line as a 'host' cell for PAR1 expression, in order to evaluate the functional properties of PAR1 in a gastric carcinoma cell background. As shown in Figure 1B, transfection of MKN45 cells with pcDNA3.1-PAR1 bearing the human full PAR1 coding sequence and a neomycin-resistance gene yielded a receptor-expressing MKN45 cell line, selected in the presence of G418.

### PAR1-expressing MKN45 cells proliferate in response to a PAR1-activating peptide and α-thrombin

**Figure 2:**
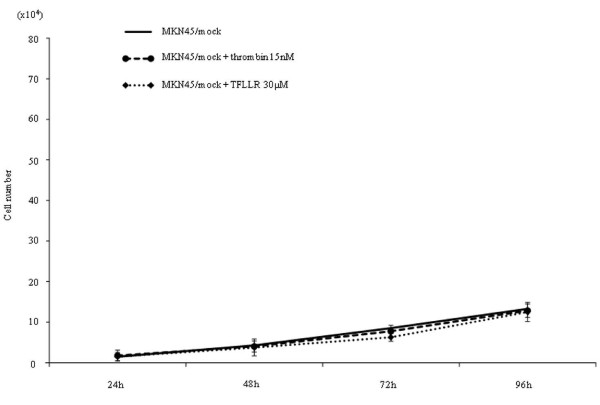
**Proliferation assay in MKN45/Mock**. This figure showed that the MKN45/mock (1.0×10^4 ^cells/well) 96 hr after adding 15 nM α-thrombin or 30 μM TFLLRN-NH_2_, specific PAR1 agonist peptide, had not significantly increased the number.

**Figure 3:**
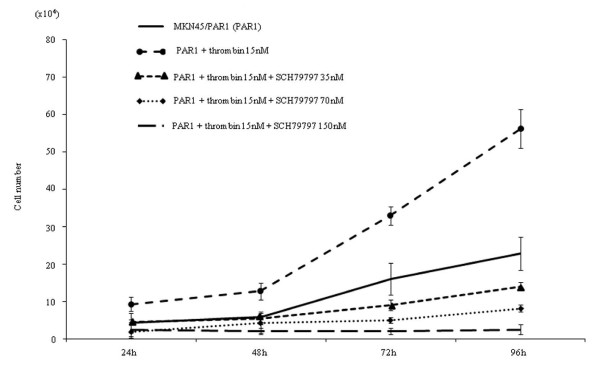
**Proliferation assay in MKN45/PAR1 with α-thrombin stimulation**. MKN45/PAR1 (1.0×10^4 ^cells/well) 96 hr after adding 15 nM α-thrombin had significantly increased the number. SCH79797, selective PAR1 antagonist, significantly reversed the growth rate of MKN45/PAR1 at 35 and 70 nM and completely abolished thrombin dependent growth progression at 150 nM in the presence of 15 nM α-thrombin.

**Figure 4:**
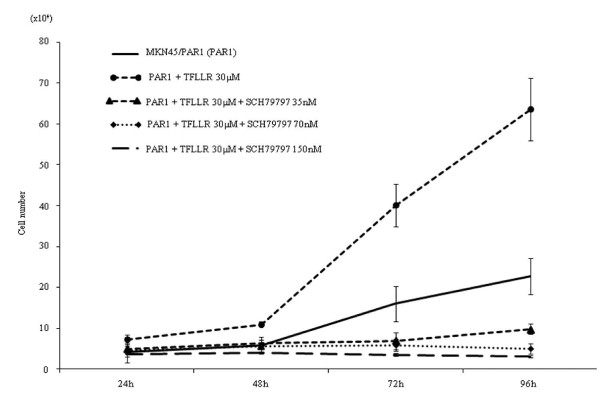
**Proliferation assay in MKN45/PAR1 with TFLLR-NH_2_**. In the same way, MKN45/PAR1 adding 30 μM TFLLR-NH_2 _had significantly increased the number, too. And SCH79797 siginificantly reversed the growth rate of MKN45/PAR1 in the presence of 30 μM TFLLR-NH_2_. (Date were expressed as mean values ± SD from triplicate experiments.)

α-thrombin clearly stimulated replication of MKN45/PAR1 over a 4 day time frame, with a greater than 2-fold increase in cell number relative to non-thrombin-treated MKN45/PAR1 at 96 hr (Figure 3). The proliferation of MKN45/mock was not stimulated either by α-thrombin or TFLLR-NH_2 _(Figure 2). Even in the absence of α-thrombin, MKN45/PAR1 outgrew the MKN45/mock at 96 hr, suggesting the presence of receptor-activating proteinases in the growth medium. Similarly, TFLLR-NH_2 _triggered an approximately 3-fold increase in cell number, in treated compared with untreated MKN45/PAR1 (Figure 4). Of particular significance, the PAR1-selective antagonist, SCH79797, in a concentration-dependent manner, was able to block the proliferative actions of both α-thrombin and TFLLR-NH_2 _(Figures 3 and 4). The data thus indicated a PAR1-specific response of MKN45/PAR1 both to α-thrombin and TFLLR-NH_2_, demonstrating the presence of a functional receptor in these cells.

### PAR1 activation induces cell invasion

**Figure 5:**
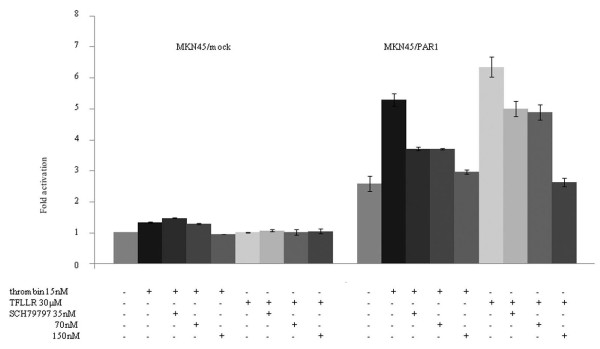
**Invasion assay in MKN45/PAR1**. Treatment of MKN45/PAR1 with 15 nM α-thrombin or 30 μM TFLLR-NH_2 _resulted in significant enhancement (5 or 6 fold increment relative to untreated control) of its invasion potential. But treatment of MKN45/mock with same conditions of α-thrombin or TFLLR-NH_2 _resulted in no significant enhancement. The inhibitory effect of the SCH79797 was clearly observed at 35 and 70 nM. There was the maximum inhibitory effect on the invasion ability of MKN45/PAR1 at 150 nM SCH79797. Basal invading activity was expressed as an absorbance of 560 nm for MKN45/mock cells which were unexposed to α-thrombin. (Date were expressed as mean values ± SD from triplicate experiments.)

MKN45/PAR1 stimulated either by α-thrombin or by TFLLR-NH_2 _showed significant acceleration of invasion (Figure 5), whereas invasion of MKN45/mock was not stimulated by either agonist (Figure 5). In regards to the proliferative response, the PAR1 antagonist SCH79797 inhibited invasion triggered by both PAR1 agonists (Figure 5). This result further supports that the α-thrombin-mediated responses in MKN45/PAR1 were due to PAR1 activation.

### PAR1-transfected MKN45 cells mimic features of PAR1-expressing gastric cancer MKN28 cells

**Figure 6:**
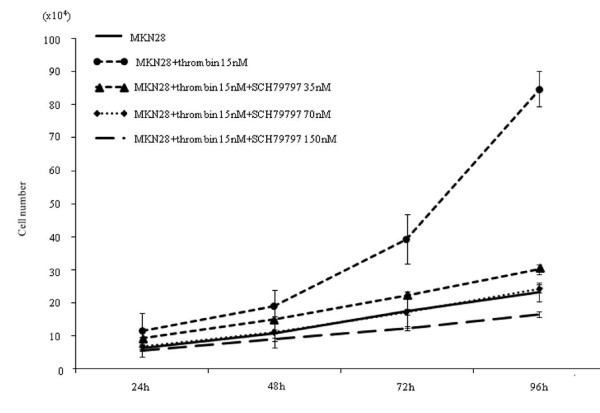
**Proliferation assay in MKN28**. The effect of α-thrombin stimulation on cellular features of MKN28 cells intrinsically expressing PAR1_. _This figure shows increases in cell proliferation rate of MKN28 cells when stimulated with 15 nM α-thrombin and the preventative effect of a PAR1-antagonist, SCH79797, on the MKN28 cell proliferation. The assay was carried out according to the same procedure as that described in Figure 2 legend.

**Figure 7:**
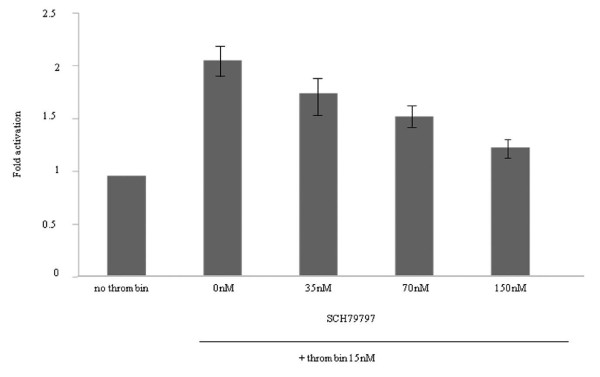
**Invasion assay in MKN28**. This shows elevated invasion functions of MKN28 cells caused by thrombin stimulation and the concentration-dependent prevention effect of a PAR1-antagonist, SCH79797. (Date were expressed as mean values ± SD from triplicate experiments.)

To compare MKN45/PAR1 with gastric cancer cells that express endogenous PAR1 mRNA and PAR1 protein, we also performed cell proliferation and invasion assays with MKN28 cells, which express PAR1 (Figure 1A &1B). Upon exposure to α-thrombin, both MKN28 cells and MKN45/PAR1 cells presented an increase in cell proliferation and invasion, and SCH79797 blocked the effects of α-thrombin (Figure 6 &7). Thus, PAR1 in both MKN45/PAR1 and MKN28 was activated by α-thrombin, resulting in cell proliferation and invasion.

### PAR1 activation induces NF-κB activation

**Figure 8:**
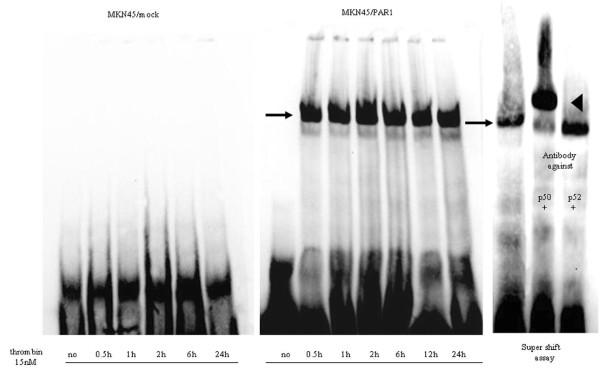
**Activation of NF-κB assessed by EMSA analysis in MKN45/mock and MKN45/PAR1 by α-thrombin stimulation**. The gel mobility shift assay was performed on the whole nuclear extract from MKN45/mock and MKN45/PAR1 (in a 12 cm diameter dish) that were incubated in the presence of 15 nM α-thrombin for 0.5, 1, 2, 6, and 24 hr. MKN45/mock samples showed no retardation bands but only intense broad signal due to biotinylated NF-κB cis-element ds-oligo probe on the gel. An arrow on left side of the middle figure indicates the NF-κB/DNA binding complex. In the super-shift assay, antibodies (2 μg) raised against NF-κBp50 and NF-κBp52 were mixed with nuclear extract obtained from PAR_1_-transfectanted cells treated with 15 nMα-thrombin for 0.5 hr. A super-shifted band was clearly detected with NF-κBp50 antibody as marked by an arrowhead, but not detected with NF-κBp52 antibody.

Having established the ability of PAR1 activation to stimulate both proliferation and invasion of MKN45/PAR1, we next evaluated the ability of PAR1 activation to stimulate transcriptional events. The EMSA showed that the PAR1 agonist α-thrombin can induce the activation of NF-κB within 30 min of treatment of the MKN45/PAR1. The EMSA signal persisted for 24 hr (Figure 8). MKN45/mock and MKN45/PAR1 not exposed to α-thrombin did not present any retarded EMSA bands (Figure 8). We performed a super-shift assay (Figure 8). In the presence of the p50-targeted antibody (but not for the p52-targeted reagent), there was a decrease in intensity of the more rapidly migrating EMSA signal, with a concomitant increase in intensity of the 'super-shifted' band (Figure 8). The ability of α-thrombin to trigger a PAR1-mediated activation of NF-κB was thereby verified.

### Dynamism of mRNA expression of NF-κB target genes induced by α-thrombin in PAR1-expressing cells

**Figure 9:**
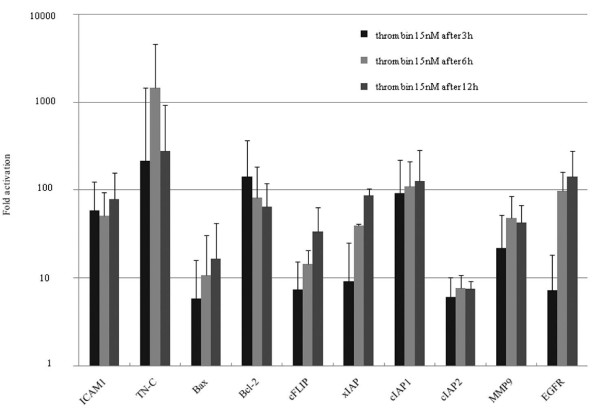
**The mRNA expression of NF-κB target genes by quantitative RT-PCR**. We selected 10 target genes of NF-κB that are concerned with cell growth and invasion. Most of the mRNA expression were increased after 15 nM α-thrombin stimulation. The mRNA expression levels of TN-C, Bcl-2, cIAP1 and EGFR reached more than 100 times the relative levels of the control values. Especially, TN-C mRNA expression level was increased. The kinetics of mRNA expression following α-thrombin stimulation in MKN45/PAR1 are different depending upon type of genes tested. The bar height indicates the calculated fold activation values for each time after stimulation with 15 nM α-thrombin. It was based on the condition of no α-thrombin stimulation. (Date were expressed as mean values ± SD from triplicate experiments.)

**Figure 10:**
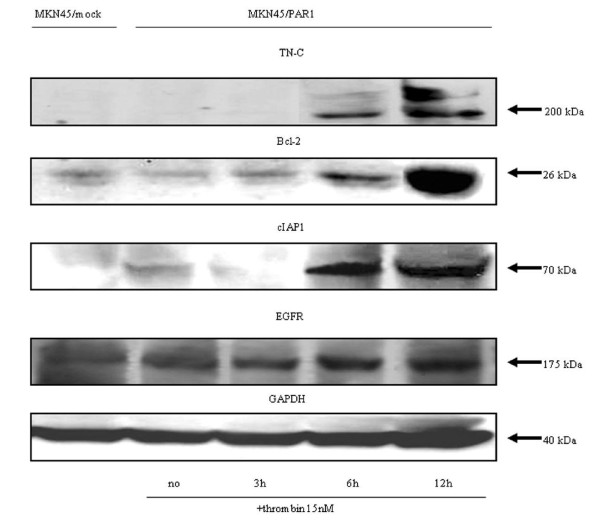
**Detection of the proteins, which were high expression mRNA under α-thrombin, by immunoblotting**. MKN45/PAR1 were stimulated with 15 nM α-thrombin for 3, 6, and 12 hr and harvested for preparing whole cell lysates. The clear extracts containing 100 μg protein was developed on SDS-PAGE. The expression of TN-C, Bcl-2, cIAP1, EGFR and GAPDH protein, which was a house keeping gene product as a control, were detected by Western blotting using specific antibodies against respective proteins (The final dilutions of TN-C, Bcl-2, cIAP1 and EGFR antibodies were 1:500, GAPDH was 1:2000). The MKN45/PAR1 were harvested at 3, 6 and 12 hr after 15 nM α-thrombin stimulation and whole cells were lysed using LIPA buffer. Significant EGFR protein expression was detected in MKN45/mock and unstimulated MKN45/PAR1. The expression above basal level was clearly observed for TN-C, Bcl-2, cIAP1 and EGFR proteins 6 hr after addition of 15 nM α-thrombin.

**Figure 11:**
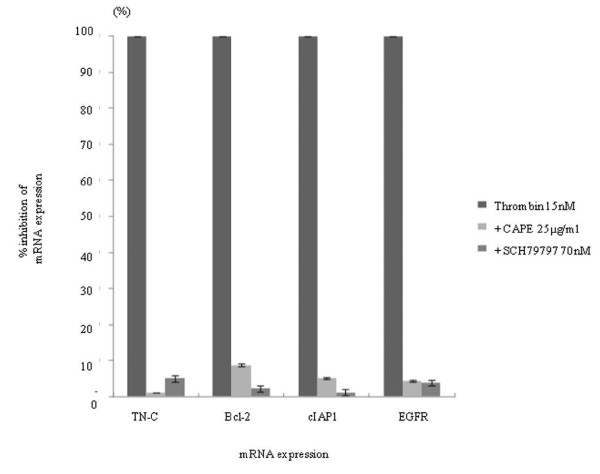
**Effect of NF-κB inhibitor CAPE and a PAR1 antagonist SCH79797 on trans-criptional activation by thrombin stimulation of NF-κB target genes**. NF-κB inhibitor or PAR1 antagonist was added at 25 μg/ml or 70 nM, respectively, to the culture medium with 15 nM α-thrombin. Six hour later, the treated cells were harvested for preparing total RNA, which was subjected to quantification for mRNA expression of four NF-κB target genes, such as, TN-C, Bcl-2, cIAP1 and EGFR by real time RT-qPCR. Each mRNA expression level after 6 hr thrombin stimulation is 100%, the suppressive effect of inhibitor or antagonist was expressed as % inhibition. (Date were expressed as mean values ± SD from triplicate experiments.)

We next evaluated the spectrum of NF-κB target genes that might be activated by α-thrombin in MKN45/PAR1. For this purpose, we selected 10 target genes of NF-κB. A-thrombin treatment (15 nM, 3 hr) caused an increase in mRNA expression levels for all NF-κB target genes tested, especially for TN-C, Bcl-2, cIAP1, and EGFR (Figure 9). The levels of TN-C, Bcl-2, cIAP1, and EGFR mRNA expression reached more than 100 times the corresponding levels of the control values in MKN45/PAR1. The increases in mRNA for these four NF-κB-stimulated genes were mirrored by increases in the levels of the four corresponding proteins, as detected by western blot analysis (Figure 10). When PAR1 activation was inhibited by SCH79797 or when NF-κB activation was inhibited by the CAPE (25 μg/ml), there was a substantial inhibition of the α-thrombin-triggered increase in the mRNA for these four NF-κB target genes in MKN45/PAR1 (Figure 11).

### High molecular-weight factors resulting from PAR1 activation can activate EGFR in an autocrine manner

**Figure 12:**
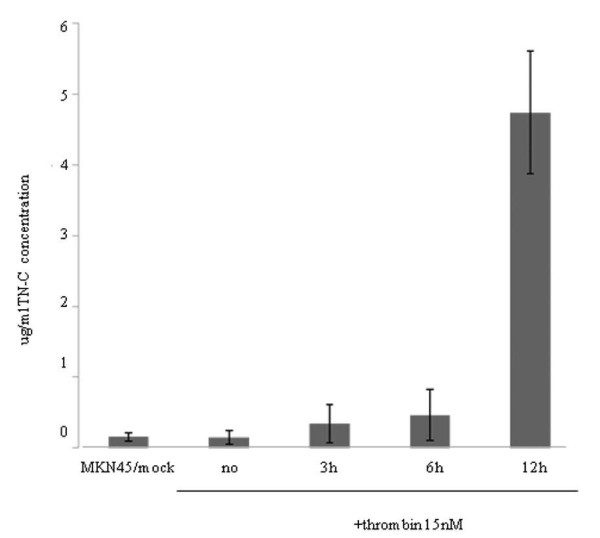
**TN-C concentration determined by ELISA in the conditioned medium of α-thrombin stimulated MKN45/PAR1**. Six cm diameter dishes with 50% confluency of MKN45/mock, MKN45/PAR1 were used for preparing the conditioned medium. α-thrombin was added at 15 nM to 6 dishes with MKN45/PAR1, and the conditioned medium was collected at indicated time (3, 6, and 12 hr) and cleared supernatant by centrifugation was subjected to ELISA assay which can detect only high molecular TN-C. The concentration of high molecular TN-C in the conditioned medium was expressed as μg/ml. (Date were expressed as mean values ± SD from triplicate experiments.)

**Figure 13:**
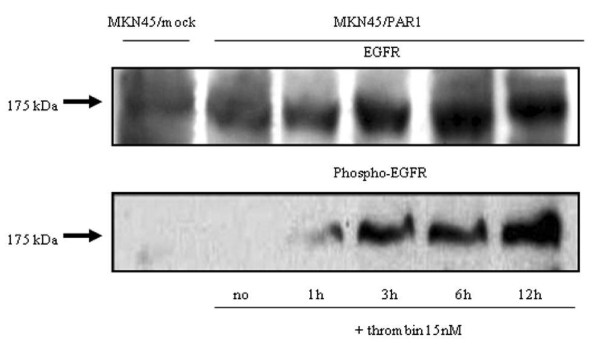
**Increases in EGFR protein expression and phosphorylation of EGFR at Tyr1173 in MKN45/PAR1 stimulated with α-thrombin**. MKN45/PAR1 were stimulated with 15 nM α-thrombin for 1, 3, 6, and 12 hr and harvested for preparing whole cell lysates. The clear extracts containing 100 μg protein was developed on SDS-PAGE. We detected by western blotting experiment using an antibody raised against total EGFR (dilution of 1: 500) was increased 3 hr after addition of 15 nM α-thrombin to MKN45/PAR1. But we detected using an antibody raised against phosphotyrosine (Tyr1173) that tyrosine phosphorylation of EGFR (dilution of 1:500) was slightly increased 1 hr after addition of 15 nM α-thrombin, and following 3 hr, phospho-EGFR was dramatically increased.

We took notice of TN-C, because its mRNA expression presented the greatest magnitude of increase by PAR1 activation. ELISA results showed that the quantity of TN-C in the culture medium increased gradually up to 3 hr after the addition of α-thrombin and that the maximum level was detected 12 hr after α-thrombin treatment in MKN45/PAR1 (Figure 12). A detectable increase in EGFR protein was also observed 6 hr after the addition of α-thrombin, and increased tyrosine-phosphorylation of the EGFR was clearly detected following 12 hr of stimulation by α-thrombin in MKN45/PAR1 (Figure 13).

**Figure 14:**
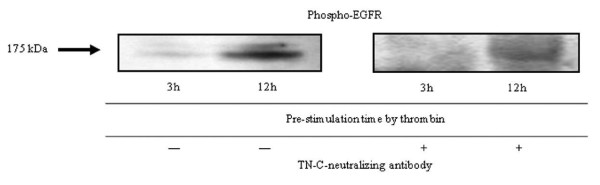
**Detection of phosholyrated EGFR by TN-C**. The conditioned medium of PAR1/MKN45 culture pre-stimulation by 15 nM α-thrombin for 3 and 12 hr was collected and partially purified by repeated concentration using ultra-filtration spin columns able to cutoff macromolecules below 200 kDa. MKN45/PAR1 were incubated either with the concentrated conditioned medium for 6 hr, which were pre-stimulated for 3 hr or 12 hr by 15 nM α-thrombin. These whole lysates of MKN45/PAR1 treated with the conditioned medium pre-stimulated for 12 hr by 15 nM α-thrombin was detected remarkably enhanced tyrosine phospholyration of the EGFR, but these whole lysates of MKN45/PAR1 with the conditioned medium pre-stimulated for 3 hr by 15 nM α-thrombin was not detected the tyrosine phospholyration of the EGFR (left). The whole lysates of MKN45/PAR1 treated with the concentrated conditioned medium with TN-C-neutralizing antibody (25 μg/ml), which was pre-stimulated for 12 hr by 15 nM α-thrombin, show no-increased in phosphor-EGFR protein quantity (right).

We hypothesized that the increased activation/phosphorylation of the EGFR might be due to the autocrine action of the increased TN-C or of other high molecular-weight constituents produced by the cells. To test this hypothesis, two separate cultures of MKN45/PAR1 cells were exposed to α-thrombin, one for a period of 3 hr and the other for a period of 12 hr, and high molecular weight proteins were isolated and concentrated from each of the resultant conditioned mediums. Subsequent cultures of MKN45/PAR1 were incubated for 6 hr with one of each of the resultant concentrates and activation/phosphorylation of the EGFR was quantified and compared. The lysates from the MKN45/PAR1, which were incubated with concentrate (deficient in proteins smaller than 200 kDa) derived from the 12 hr α-thrombin stimulated conditioned medium presented an increase in phospho-EGFR, but the lysates from the MKN45/PAR1 which were cultured with the concentrate derived from the 3 hr α-thrombin stimulated conditioned medium presented no detectable bands (Figure 14 left). The level of TN-C was minimal in the concentrate derived from the 3 hr α-thrombin stimulated conditioned medium, but abundant in the concentrate derived from the 12 hr α-thrombin stimulated conditioned medium. These results clearly indicate that α-thrombin-stimulated MKN45/PAR1 produced high-molecular-weight constituents (possibly, TN-C), which can activate EGFR in an autocrine manner.

**Figure 15:**
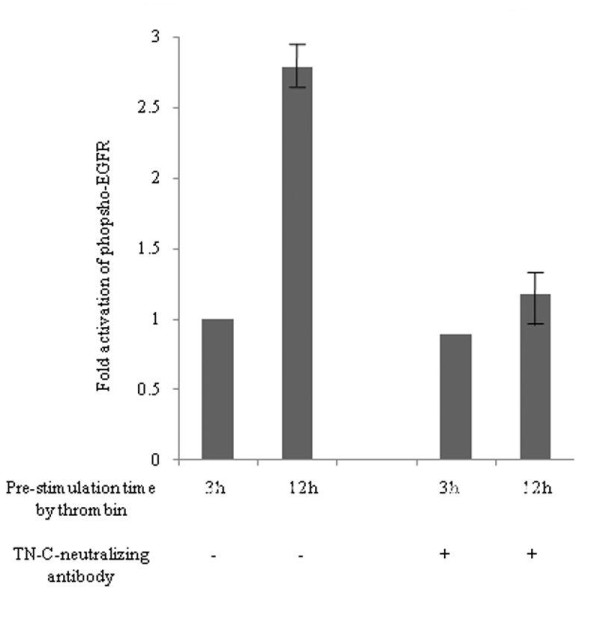
**Phospho-EGFR signal quantitatively diminished in the presence of TN-C-neutralizing antibody by histograms**. The phospho-EGFR signal quantitatively diminished in the presence of TN-C-neutralizing antibody by histograms. The bar height indicates the calculated fold activation values. It was based on the condition with the concentrated conditioned medium, which was obtained after at 3 hr 15 nM α-thrombin stimulation with or without TN-C-neutralizing antibody. Data are expressed as intensity units obtained by densitometric analysis. (Date were expressed as mean values ± SD from triplicate experiments.)

We suspected that TN-C would be present along with constituents with sizes greater than 200 kDa. To determine if TN-C might be responsible for the ability of the concentrated medium to activate EGFR, we repeated the experiment described above in the presence of a TN-C-neutralizing antibody. The antibody markedly reduced the ability of the high-molecular-weight cutoff medium to stimulate the phosphorylation of the EGFR (Figure 14 right). And we showed by means of histograms that phosphor-EGFR signal quantitatively diminished in the presence of TN-C-neutralizing antibody (Figure 15). The data indicates that TN-C can contribute in an autocrine manner to stimulate phosphorylation of EGFR.

## Discussion

The main finding of our study is that activation of PAR1 triggers activation of NF-κB and EGFR for a long period, and TN-C, which is overexpressed by PAR1 activation, may be associated with EGFR activations. Our data now show not only that the histological presence of PAR1 is correlated with the pathological findings associated with invasion and metastasis in gastric cancer [22], but also that this receptor and its activating proteinases, including thrombin and other serine proteinases [8] can be seen as mechanistically important factors driving the process of gastric cancer cell proliferation and invasion.

Because our work used a PAR1 null cell as the host for PAR1 expression, our data clearly demonstrate the oncogenic potential of PAR1 itself in a gastric cancer cell background, apart from factors other than PAR1 that can confer the oncogenic phenotype. Both the absence of α-thrombin effects in the PAR1 null cells and the ability of the PAR1-selective antagonist, SCH79797, to block the actions of α-thrombin in MKN45/PAR1 indicate that the actions of α-thrombin were due to PAR1 activation and not to other α-thrombin targets, like PAR4 or triggering by metalloproteinases [12].

Our qPCR analysis of the spectrum of NF-κB target genes up-regulated by PAR1 activation revealed quite a number of proteins, for example TN-C, Bcl-2 and cIAP1, for which up-regulation has been previously associated with a tumorigenic phenotype (Figure 9&10). The prolonged time frame over which the mRNAs levels are elevated after α-thrombin stimulation (up to 12 hr) implies that signals in addition to the one triggered by PAR1 may be involved. For instance, the direct activation of PAR1 by α-thrombin or PAR1-activating peptide would be expected to be down-regulated over a relatively short time frame, as can be observed for the stimulation of intracellular calcium transients [29,30] or for the activation of Mitogen-activated protein kinase (MAPK) (often maximal at 5 minutes, declining to baseline within 1 hr). Yet, in contrast with a PAR1-activating peptide, α-thrombin as an agonist is known to cause a prolonged activation of MAPK and enhanced mitogenesis [31]. It has been hypothesized that these long-term actions of α-thrombin, in contrast with the effects of PAR-activating peptides may be mediated by receptors and mechanisms other than those encompassing PAR1 [32]. Thus, although we also have shown that NF-κB and EGFR activation initially were triggered by activation of PAR1 in the early phase (Figures 8 &13) [19,33] the sustained responses very likely are mediated by 'feed-forward' mechanisms, possibly involving the production of autocrine stimulatory factors like the one(s) detected in the concentrated cell supernatants and/or the sequential and synergistic cooperation of several transcription factors in addition to NF-κB. This sequence of events set in motion by PAR1 activation may reflect a generalized 'oncogenic signal matrix' that may be initiated by a variety of mitogenic agents like thrombin.

Apart from cell-derived proteinases as potential autocrine/paracrine factors, our work points to the possible autocrine importance of secreted TN-C that was observed to be a PAR1-induced gene and that could be recovered in concentrate of conditioned medium of cultures of α-thrombin-treated MKN45/PAR1 (RT-PCR data; ELISA assay and identification by mass spectroscopy). PAR1 activation accounted for α-thrombin-induced tyrosine phospholyration of EGFR in renal carcinoma cells [20]. We also confirmed that the EGFR itself was elevated in response to PAR1 activation in gastric carcinoma cells (Figures 9, 10 &13). Since the EGF-like sequence repeats derived from TN-C can act as agonists for the EGFR in terms of MAPK activation [34], it is tempting to speculate that secreted TN-C might act as an autocrine activator of the EGFR to enhance the mitogenic effect of PAR1 activation.

Further, the high-molecular-weight fractions recovered from the conditioned medium of α-thrombin-stimulated MKN45/PAR1 cultures were able to enhance the phosphorylation of the EGFR (Figure 14 &15), in keeping with the hypothesis that the α-thrombin-stimulated cells can produce autocrine factors that can activate the EGFR. That TN-C itself represents that factor is an open question, since even the TN-C derived EGF repeat sequences do not significantly trigger EGFR autophosphorylation, although they do trigger EGFR-mediated activation of MAPK [34]. Further, it is not yet known if the EGF-like repeats in TN-C can activate the EGFR when present in the intact TN-C sequence. Notwithstanding, the high-molecular-weight fraction from the MKN45/PAR1 concentrates were able to induce EGFR phosphorylation, and the TN-C-targeted-neutralizing antibody significantly reduced that effect (Figure 14 &15). The data thus imply an autocrine role for TN-C in cancer cells that clearly merits further work to elucidate the mechanism. TN-C, an adhesion modulatory extracellular matrix molecule, is implicated in signal transduction, proliferation and invasion in various cancers [35-38]. Our results showed that TN-C was involved in the PAR1-mediated EGFR transactivation in cancer cells for the first time.

## Conclusion

Finally, we showed that the signaling pathways that responded to PAR1 activation involving the activation of NF-κB and transactivation of EGFR, which might be stimulated by TN-C, resulted in an increase in gastric cancer cell proliferation and invasion. These data indicate that PAR1 is deeply associated with gastric cancer progression, and thus a very attractive novel therapeutic target for blocking the progression of invasive and metastatic gastric cancers.

## Competing interests

The authors declare that they have no competing interests.

## Authors' contributions

DG performed all experiments, analyzed the data and drafted manuscript. YH participated in the study design, data interpretation and scientific revision of the manuscript. TG provided molecular genetic advice. KK carried out statistical advice. SM participated in study design and provided molecular genetic advice. AY participated in scientific revision of the manuscript. All authors read and approved the final manuscript.

## Pre-publication history

The pre-publication history for this paper can be accessed here:

http://www.biomedcentral.com/1471-2407/10/443/prepub
